# Linking Patient Encounters across Primary and Ancillary Electronic Health Record Systems: A Comparison of Two Approaches

**DOI:** 10.1055/s-0044-1782679

**Published:** 2024-04-10

**Authors:** Marcos A. Davila, Evan T. Sholle, Xiaobo Fuld, Mark L. Israel, Curtis L. Cole, Thomas R. Campion

**Affiliations:** 1 Information Technologies & Services Department, Weill Cornell Medicine, New York, New York, United States; 2 Department of Population Health Sciences, Weill Cornell Medicine, New York, New York, United States; 3 Clinical IT Shared Services, NewYork-Presbyterian, New York, New York, United States; 4 Department of Medicine, Weill Cornell Medicine, New York, New York, United States; 5 Clinical and Translational Science Center, Weill Cornell Medicine, New York, New York, United States; 6 Department of Pediatrics, Weill Cornell Medicine, New York, New York, United States

**Keywords:** secondary use, encounters, record linkage, data warehouse, data mart

## Abstract

**Background** To achieve scientific goals, researchers often require integration of data from a primary electronic health record (EHR) system and one or more ancillary EHR systems used during the same patient care encounter. Although studies have demonstrated approaches for linking patient identity records across different EHR systems, little is known about linking patient encounter records across primary and ancillary EHR systems.

**Objectives** We compared a patients-first approach versus an encounters-first approach for linking patient encounter records across multiple EHR systems.

**Methods** We conducted a retrospective observational study of 348,904 patients with 533,283 encounters from 2010 to 2020 across our institution's primary EHR system and an ancillary EHR system used in perioperative settings. For the patients-first approach and the encounters-first approach, we measured the number of patient and encounter links created as well as runtime.

**Results** While the patients-first approach linked 43% of patients and 49% of encounters, the encounters-first approach linked 98% of patients and 100% of encounters. The encounters-first approach was 20 times faster than the patients-first approach for linking patients and 33% slower for linking encounters.

**Conclusion** Findings suggest that common patient and encounter identifiers shared among EHR systems via automated interfaces may be clinically useful but not “research-ready” and thus require an encounters-first linkage approach to enable secondary use for scientific purposes. Based on our search, this study is among the first to demonstrate approaches for linking patient encounters across multiple EHR systems. Enterprise data warehouse for research efforts elsewhere may benefit from an encounters-first approach.

## Introduction

Clinical and translational scientists need patient data from electronic health record (EHR) systems to conduct research. 1 Although academic medical centers increasingly use a single enterprise EHR application to support multiple clinical and billing functions, 2 many health care organizations have deployed a “best of breed” approach connecting a primary EHR system with one or more ancillary EHR systems used in specific care settings (e.g., cardiology and anesthesiology) through automated interfaces that exchange patient and encounter records along with details of care. 3 Care team members often document in primary and ancillary EHR systems for a patient during the same episode of care. To effectively use real-world data from EHR systems to generate real-world evidence, informatics professionals often transform raw clinical data used to support individual patient care transactions into “research-grade” or “research-ready” data used to analyze populations of patients. 4 5 Whether an EHR-derived dataset is “fit for purpose” depends on multiple factors, including accuracy and completeness of patient and encounter records as a prerequisite to understanding clinical details. Although studies have demonstrated approaches for linking patient identity records across disparate EHR systems to enable care and research, 6 7 8 9 10 11 12 13 to the best of our knowledge no studies have described approaches for linking patient encounter records across multiple EHR systems, including primary and ancillary systems used during the same patient care encounter.

## Objectives

Our motivation for investigating encounter record linkage stemmed from data quality issues observed when integrating data from our institution's primary EHR system and an ancillary EHR system used in perioperative settings. Specifically, our goal was to integrate elements from the primary EHR, such as laboratory results and procedure codes, with anesthesia-specific elements from the ancillary EHR, such as the use of nasal cannula. In querying an initial version of a data mart combining primary and ancillary EHR system data, clinicians indicated counts of patients meeting inclusion criteria were substantially lower than anticipated, suggesting that our initial approach did not properly integrate data from the main and ancillary EHR systems. Undergirding our initial approach were two assumptions. First, patient and encounter record identifiers exchanged by the two EHR systems through automated interfaces represented both clinically useful and research-ready data. Second, modeling data from the EHR systems should occur with patients first, encounters second, and clinical details (e.g., vital signs, laboratory results) third as informed by popular clinical extract–transform–load approaches. 14 15 Observing that a patients-first approach failed to properly integrate data from primary and ancillary EHR systems used in the same patient care encounter, we pursued an encounters-first approach. In this case study, we compared a patients-first approach versus an encounters-first approach for linking patient encounter records across multiple EHR systems.

## Methods

### Setting

Located in New York City, Weill Cornell Medicine (WCM) and NewYork-Presbyterian (NYP) have long shared a clinical affiliation. WCM, the biomedical research and education unit of Cornell University, employs more than 1,600 physicians as faculty members of Weill Cornell Medical College who see patients across more than 20 multispecialty practice locations in the metropolitan area. WCM physicians have inpatient admitting privileges to NYP/Weill Cornell Medical Center on Manhattan's Upper East Side.

WCM and NYP are clinical partners but separate corporations with separate information technology organizations. Historically, WCM and NYP have deployed different billing and clinical information systems to enable patient care operations. Automated interfaces connected WCM and NYP systems to synchronize data by utilizing an enterprise master patient index (EMPI) to link patient records and a billing account identifier to link encounter records. EMPI enabled interfaces to link all different representations of every patient's medical record number (MRN), a unique patient identifier (UPI) in use in certain ancillary and legacy EHR systems.

During the study period, WCM managed the primary EHR system, EpicCare (Epic Systems, Verona WI, USA), while NYP managed the ancillary EHR system used in perioperative settings, CompuRecord (Philips NV, Amsterdam NL). NYP also managed other ancillary EHR systems, including the Eagle billing system and the Allscripts Sunrise Clinical Manager (SCM) EHR system used in inpatient and emergency settings. Automated interfaces updated all systems with patient and encounter level information when billing and clinical workflows created patient identities and encounters. Notably, Eagle generated and maintained billing account identifiers used to track visits that occurred at NYP, which flowed from Eagle into Allscripts SCM and CompuRecord as well as WCM Epic. Within WCM Epic, the application associated NYP billing account identifiers with Epic encounter records. A one-to-one relationship between NYP billing account identifiers and WCM Epic encounter identifiers did not exist, as one billing account could be associated with one or more Epic encounter records.

In addition to separate management of EHR systems by WCM and NYP, a distinct organizational unit within WCM provided management of data from primary and ancillary EHR systems for research purposes. Specifically, WCM Research Informatics division operated an enterprise data warehouse containing data from multiple primary and ancillary EHR systems across WCM and NYP, provided tools and services for obtaining data, served as the honest broker of patient identity for research, 16 and performed this study.

### Study Design, Patient Population, and Data Collection

We conducted a retrospective observational study of patients with data in one ancillary EHR system during the time period of January 2010 to January 2020.

From the CompuRecord ancillary EHR system, we obtained all patient records and encounter records via an extract provided by the NYP team managing the system. Each patient record consisted of an MRN. Each encounter record consisted of a billing account identifier, MRN, internal case account number, and date of service. The billing account identifier was the unique identifier for encounter records, and MRN was the only identifier tracked for patient records.

From the Epic primary EHR system, we obtained all patient records and encounter records via query of the WCM Epic Clarity database. Each patient record consisted of an internal Epic patient identifier, MRN, and EMPI; EMPI was the unique identifier, as patients had one or more MRNs. Each encounter record consisted of one or more MRNs, an EMPI, one internal Epic encounter identifier, one billing account identifier, and one or more dates of service.

### Approaches for Matching Records

**Fig. 1:**
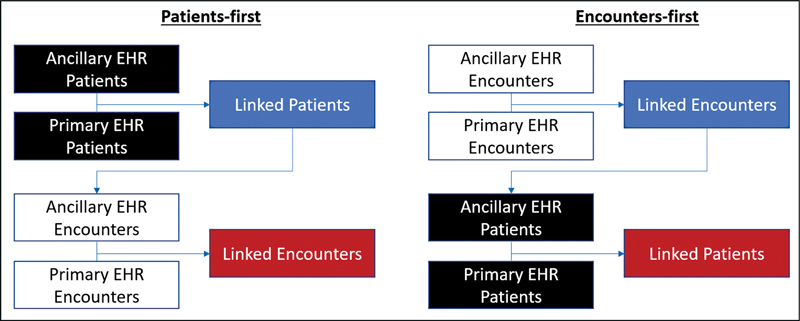
A comparison of the patients-first approach and the encounters-first approach.

Fig. 1 illustrates the general steps of the patients-first and encounters-first approaches, both of which operated deterministically rather than probabilistically. In the patients-first approach, first, for all patient records from the ancillary EHR system, we linked with patient records in the primary EHR system using the common UPI (i.e., MRN). Second, for all encounter records from the ancillary EHR system of those patient records linked in the first step, we linked with encounter records in the primary EHR system using the common unique encounter identifier (i.e., billing account number). In the encounters-first approach, first, for all encounter records from the ancillary EHR system, we linked with encounter records in the primary EHR system using the common encounter identifier (i.e., billing account number). Second, for all patient records from the ancillary EHR system of those encounter records linked in the first step, we linked with patient records in the primary EHR system using the common UPI (i.e., MRN). Setting-specific implementation details are in Supplementary Appendices 1 to 3 (available in the online version).

### Validation

Prior to evaluation and as part of development, we validated the patients-first and encounters-first approaches through a process of iterative manual review and adjustment. Because examination of all output was not feasible, we performed a manual chart review of approximately 1,000 encounters, comparing underlying database records to front-end EHR user interface displays to verify the alignment of visit, patient, and time of visit. Gaps in patient and encounter linking observed in the patients-first approach served as the impetus for comparing patients-first and encounters-first approaches to the front-end EHR. We also wrote structured query language (SQL) statements to verify that each row in the patient linking table belonged to exactly one patient and each row in the encounter linking table belonged to exactly one visit.

### Evaluation

For the patients-first approach and the encounters-first approach, we measured the number of patients and encounter links created across the primary EHR system and ancillary EHR system and runtime. To determine the number of links of patients and encounters, we calculated the number of unique records in patient and encounter mapping tables. All experiments used a Microsoft SQL Server 2016 virtual environment with 24 vCPU cores, 32 GB RAM, and 50 TB of disk space.

## Results

As shown in Table 1 , during the study period the ancillary EHR system contained records for 348,904 unique patients with 533,283 encounters. While the patients-first approach linked less than half of the patients (43%) and encounters (49%) across the ancillary EHR and primary EHR, the encounters-first approach linked nearly all patients (98%) and 100% of encounters. The runtime of the encounters-first approach was 20 times faster than the patients-first approach for linking patients and 33% slower for linking encounters.

**Table 1:** Comparison of linkages created by and runtime of approaches

	Patient records ( *n* = 348,904)	Encounter records ( *n* = 533,283)
Links created		
Patients first, *n* (%)	150,670 (43%)	259,534 (49%)
Encounters first	342,650 (98%)	533,283 (100%)
Runtime (minutes)		
Patients first	20	60
Encounters first	1	90

## Discussion

In attempting to link encounter records across a primary EHR system and an ancillary EHR system, an encounters-first approach outperformed a patients-first approach, although it was approximately 13% slower. Findings suggest that encounter record identifiers shared across primary and ancillary EHR systems may be research-ready, whereas patient record identifiers are not. Linking encounter records followed by patient records, rather than linking patient records followed by encounter records, may improve data quality and “fit for purpose” of data derived from multiple EHR systems.

A chart review confirmed that the 2% of records not linked by the encounters-first approach were attributed to missing or incorrectly captured data within the ancillary EHR system. The chart review also confirmed the validity of the linkages created by the encounters-first approach and that patients were correctly linked to their encounters. Furthermore, the chart review confirmed that if a patient's MRN in the ancillary EHR system changed across encounters, mappings of MRN-to-EMPI in the primary EHR system correctly resolved the patient's identity. Perhaps most critically, clinician review confirmed that the use of the encounters-first approach yielded a data mart with patient counts meeting expectations, which the initial patients-first approach failed to achieve.

The encounters-first approach linked more patients (98%) than the patients-first approach (43%) because the billing account numbers, the unique encounter identifiers in both systems, appeared to be maintained more consistently than MRNs, the UPIs in both systems. As described in the Appendix, MRN formatting was not standardized across the two systems. Although both the primary and ancillary EHR systems treated MRNs as string literals, the primary EHR system used a variable length of digits for the MRN but the ancillary EHR system used exactly nine characters in length. While it is possible to define logic to handle these and other cases for matching patient identifiers, the encounters-first approach demonstrated that the fidelity of encounter identifiers enabled accurate linkage of both visit and patient identity, obviating the need to implement sophisticated techniques for matching patient identifiers. Although the encounters-first approach required 13% longer runtime to account for a larger search space of encounter records compared with the patients-first approach, the tradeoff of time for quality appears justified.

The automated interfaces connecting disparate “best of breed” EHR systems in medical centers enable clinically useful display of data in front-end EHR systems for the primary purpose of patient care. To make data research-ready for secondary use, 4 documentation for popular common data models i2b2 17 18 and OMOP 18 appears to suggest modeling patients followed by encounters. For i2b2, the documentation describes the contents of the OBSERVATION_FACT table from a patients-first perspective, 14 although the SQL that powers the i2b2 web client joins on encounters before reducing to a count of patients. For OMOP, documentation indicates that patients with no clinical events should be included in the PERSON Table. 15 Additionally, the OMOP Achilles tool is designed to detect patient-specific issues but not encounter-specific issues. The phrasing of the documentation for i2b2 and OMOP suggests informatics professionals model patients before encounters. However, findings from this study suggest otherwise and that modeling encounters before patients may be critical to accurate linkage of records for the secondary use of EHR data.

**Fig. 2:**
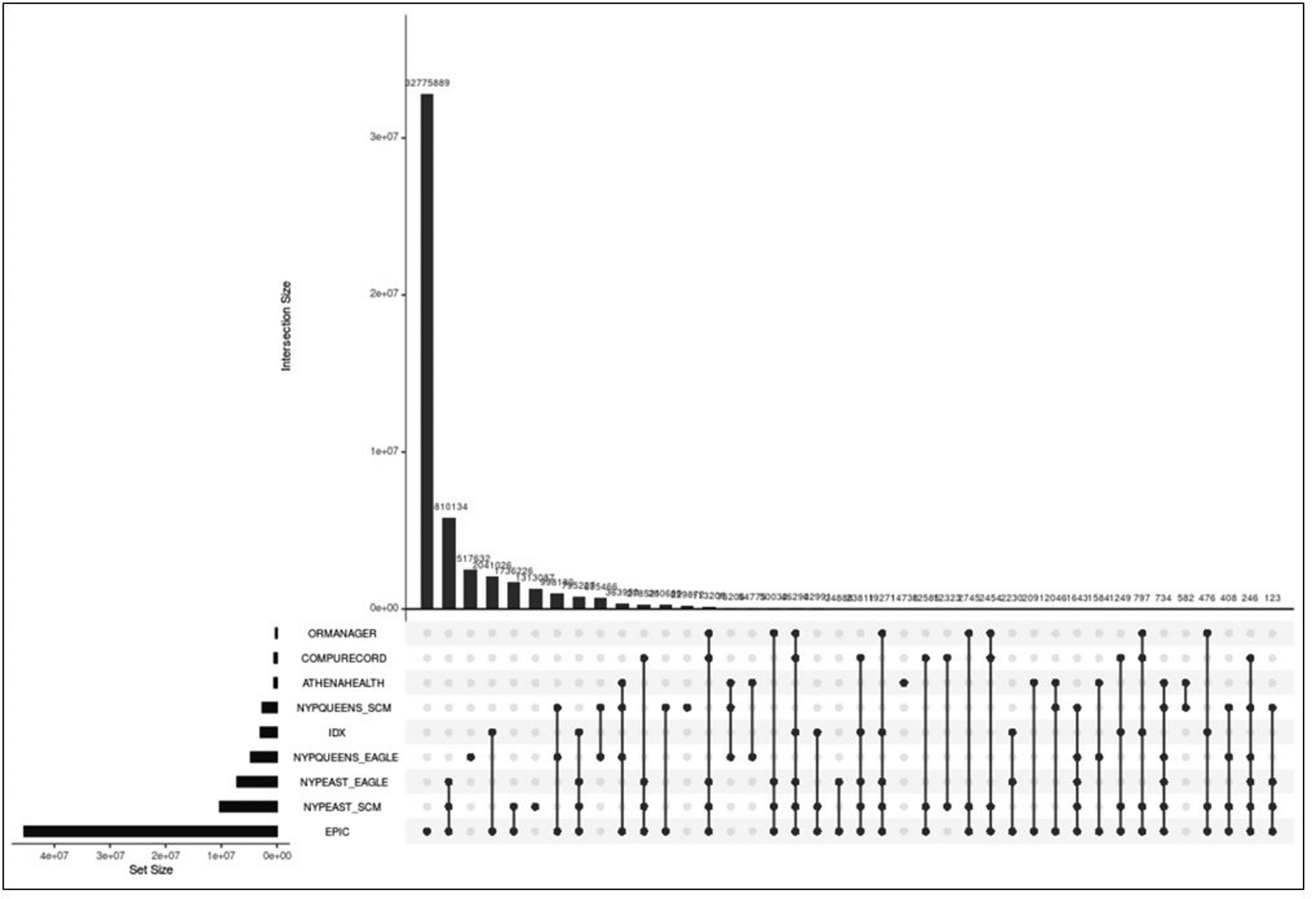
The encounters-first approach scaled up from integrating patient encounters across two EHR systems (CompuRecord & Epic) to nine systems across multiple medical center campuses.

Based on the success of experiments of the encounters-first approach with the primary and ancillary EHR systems described above, we scaled the approach to include all ancillary EHR systems used at our institution's main NYP/Weill Cornell Medical Center campus as well as the NYP/Queens-affiliated regional hospital with similar automated interfaces to WCM Epic for clinical care. As shown in Fig. 2 , the encounters-first approach facilitated the integration of data from the Allscripts SCM EHR and Eagle billing systems, which operated as separate instances for the two hospital campuses, along with clinical and billing systems both in active use (e.g., OR Manager) and retired (e.g., IDX). While some patient and encounter data are shared between the NYP/Weill Cornell Medical Center and NYP/Queens systems, most patients and encounters are unique to one campus.

Academic medical centers increasingly have adopted the function of an enterprise data warehouse for research (Enterprise data warehouse for research [EDW4R]) to aggregate, transform, and deliver EHR data to researchers to enable science. 19 20 Primary and ancillary EHR systems continue to exist despite the proliferation of an EHR monoculture 2 in certain segments of the health care economy. To leverage data documented by clinicians in one system and integrated across multiple other systems, EDW4R leaders may find valuable the approaches described in this study of a primary and ancillary EHR system extended to multiple ancillary EHR systems. EDW4R efforts elsewhere may benefit from an encounters-first approach to integrate data from primary and ancillary EHR systems.

This study has limitations. First, we conducted this study in a single institution, and it is unknown whether the phenomena observed exist elsewhere. However, the expansion of the encounters-first approach from our main hospital campus to a regional hospital campus suggests the approach can scale. Additionally, other institutions can test the hypothesis that patient and encounter records shared among EHR systems through automated interfaces are “research-grade” 4 or “research-ready.” 21 Second, although the ancillary data made available to our team included MRNs, it did not include patient names, which prevented the use of name-matching algorithms (e.g., Jaro-Winkler) to account for cases where MRNs were malformed and required secondary patient identity verification. Institutional decisions prevented sharing patient names with our team, and other institutions can benefit from working with ancillary EHR system managers to ensure the sharing of patient names along with MRN and other demographics. Third, our team, which does not manage EHR systems, integrated data from clinical and billing information systems for which we had incomplete or no documentation of implementation decisions regarding automated interface configuration. As a result, we made assumptions about data that may be incorrect, although clinical team feedback and measures in this study suggest the encounters-first approach has face validity and is a reasonable response to uncertainty. In other institutions, analytics teams may be similarly separated from transactional system teams, and lessons learned from this investigation may inform efforts to improve and share documentation about automated interfaces between clinical information systems to improve the secondary use of data. Finally, we conducted this study at an institution in the United States, where nationwide UPIs are not implemented. 22 Scholars in the United Kingdom, where each patient has a nationally unique patient identifier, have demonstrated the value of a nationwide UPI for disease surveillance. 23 Policymakers in the United States and elsewhere can potentially address patient identity linking through legislation.

## Conclusion

Generating accurate patient and encounter record linkages across disparate EHR systems connected by automated interfaces is a necessary and potentially complicated step to enable the secondary use of EHR data for research. Patient and encounter records in source systems may have local or systematic inconsistencies that are challenging to handle. Compared with a patients-first approach, an encounters-first approach was slightly slower in linking patient and encounter records. However, the encounters-first approach yielded nearly complete patient and encounter linking across primary and ancillary EHR systems. Understanding how patient and encounter identity records align across different EHR systems is necessary to enable research-ready data fit for secondary use.

## Clinical Relevance Statement

Effective secondary use of data from electronic health record (EHR) systems requires data to be research-ready rather than solely clinically useful. Because patient encounter records may be research-ready while patient identity records may not, linking encounter records before linking patient records may yield higher quality, research-ready datasets to enable science. Enterprise data warehouse for research (EDW4R) efforts may benefit from an encounters-first approach to integrate data from primary and ancillary EHR systems.
