# Supplementary material for: Linking Patient Encounters across Primary and Ancillary Electronic Health Record Systems: A Comparison of Two Approaches
Source: ACI open. Author manuscript; Available in PMC 2024 May 17. (PMC11101195; doi:10.1055/s-0044-1782679)
Supplement: Appendices [file NIHMS1985057-supplement-Appendices.pdf]

Supplementary Material

Supplementary Appendix 1 Textual description of WCM-specific implementation of patients-first and encounters-first approaches

At our institution, details of the patients-first approach included the following. First, for each patient record from CompuRecord, join Epic and CompuRecord on MRN and billing account and insert EMPI, Epic MRN, CompuRecord MRN, a NULL placeholder for a synthetic patient identifier, and related demographic elements (e.g., date of birth) into a patient mapping table. MRN can be formatted differently between Epic and CompuRecord, so both are tracked. Second, since patients can have more than one MRN, insert EMPI, any other available Epic MRNs, and demographic elements as new rows using the Epic EMPI to MRN crosswalk. Finally, we updated each row with a synthetic patient identifier using a SQL SEQUENCE, which generated ascending numeric values based on EMPI. This completes the creation of the patient mapping table. To create the encounter mapping table, first join Epic and CompuRecord on MRN and insert EMPI, Epic contact number, Epic and CompuRecord dates, billing account identifier, a NULL placeholder for a synthetic encounter identifier, and MRN. Second, insert all Epic only encounters for the cohort. Finally, we used a separate sequence to update the table with a synthetic encounter identifier. This identifier was based on billing accounts first, Epic contacts second (►**Appendix 2**).

At our institution, details of the encounters-first approach included the following. First, for each patient record from CompuRecord, we performed a SQL CROSS JOIN of EMPI to every Epic MRN using Epic's EMPI to MRN crosswalk. For every combination of EMPI and Epic MRN, a LEFT JOIN between Epic and CompuRecord is used to bridge encounters on MRN and billing account when available. EMPI, Epic MRN, CompuRecord MRN, any available START_DATEs and END_DATEs, billing account number, Epic contact number, and two NULL columns for the synthetic patient identifier and the synthetic encounter identifier are inserted into an encounter mapping table. Notably, the CROSS JOIN related one record from one table to every record in another table, which created a Cartesian product. This product is required to address cases where Epic and CompuRecord use different MRNs to refer to the same patient and billing encounter. Second, we used a SQL SEQUENCE to generate the synthetic patient identifier based on MRN. Third, we used a separate SQL SEQUENCE to generate a synthetic encounter identifier. This sequence populates the encounter mapping table with an identifier based on billing account first. At this stage, there exist encounters in the table that are assigned an identifier and NULL because the CROSS JOIN from the first step created unused combinations of EMPI and MRN. These combinations

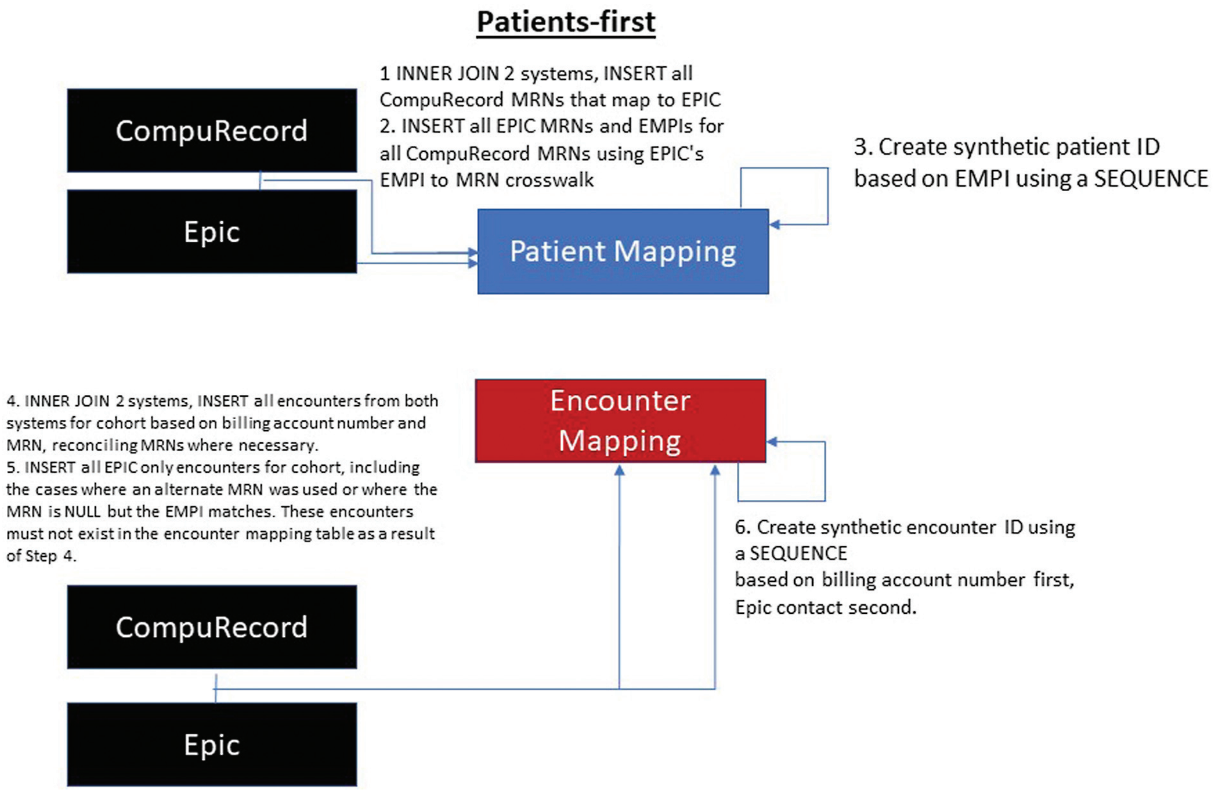

Supplementary Appendix 2 Illustration of WCM-specific implementation of patients-first approach.

 © 2024. The Author(s).

with NULL identifiers should be updated to the identifier of the matching billing account. It is required to harmonize these rows before performing the fourth step, which is using the sequence to assign identifiers based on Epic contact number. Otherwise, these unused combinations will assign multiple identifiers to one encounter. Finally, we inserted the unique combination of EMPI, MRN, Epic patient ID, and the synthetic patient identifier into a patient mapping table (►Appendix 3).

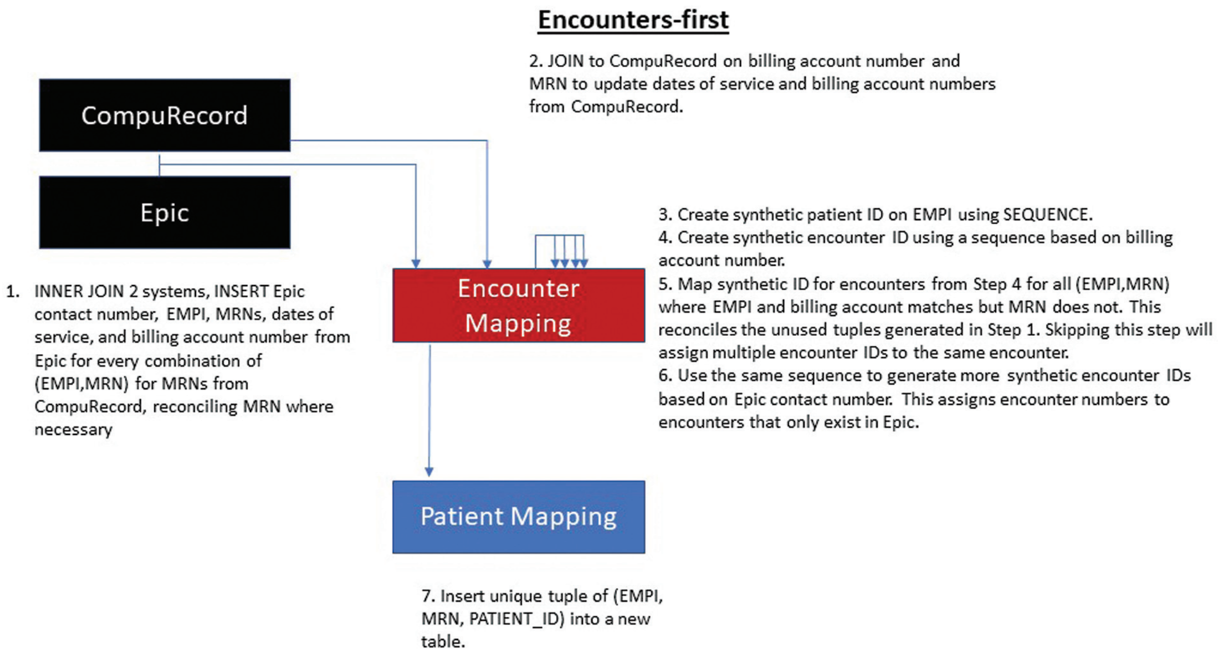

Supplementary Appendix 3 Illustration of WCM-specific implementation of encounters-first approach.

 © 2024. The Author(s).
